# Sensitive detection of mitochondrial DNA variants for analysis of mitochondrial DNA-enriched extracts from frozen tumor tissue

**DOI:** 10.1038/s41598-018-20623-7

**Published:** 2018-02-02

**Authors:** M. J. A. Weerts, E. C. Timmermans, R. H. A. M. Vossen, D. van Strijp, M. C. G. N. Van den Hout–van Vroonhoven, W. F. J. van IJcken, P. J. van der Zaag, S. Y. Anvar, S. Sleijfer, J. W. M. Martens

**Affiliations:** 1 Department of Medical Oncology and Cancer Genomics Netherlands, Erasmus MC Cancer Institute, Rotterdam, The Netherlands; 2 Philips Research Laboratories, High Tech Campus 11, 5656 AE Eindhoven, The Netherlands; 3 Leiden Genome Technology Center (LGTC), Department of Human Genetics, Leiden University Medical Center, Leiden, The Netherlands; 4 Center for Biomics, Erasmus MC, Rotterdam, The Netherlands; 5 Department of Human Genetics, Leiden University Medical Center, Leiden, The Netherlands; 6 Department of Clinical Pharmacy and Toxicology, Leiden University Medical Center, Leiden, The Netherlands

## Abstract

Large variation exists in mitochondrial DNA (mtDNA) not only between but also within individuals. Also in human cancer, tumor-specific mtDNA variation exists. In this work, we describe the comparison of four methods to extract mtDNA as pure as possible from frozen tumor tissue. Also, three state-of-the-art methods for sensitive detection of mtDNA variants were evaluated. The main aim was to develop a procedure to detect low-frequent single-nucleotide mtDNA-specific variants in frozen tumor tissue. We show that of the methods evaluated, DNA extracted from cytosol fractions following exonuclease treatment results in highest mtDNA yield and purity from frozen tumor tissue (270-fold mtDNA enrichment). Next, we demonstrate the sensitivity of detection of low-frequent single-nucleotide mtDNA variants (≤1% allele frequency) in breast cancer cell lines MDA-MB-231 and MCF-7 by single-molecule real-time (SMRT) sequencing, UltraSEEK chemistry based mass spectrometry, and digital PCR. We also show *de novo* detection and allelic phasing of variants by SMRT sequencing. We conclude that our sensitive procedure to detect low-frequent single-nucleotide mtDNA variants from frozen tumor tissue is based on extraction of DNA from cytosol fractions followed by exonuclease treatment to obtain high mtDNA purity, and subsequent SMRT sequencing for (*de novo*) detection and allelic phasing of variants.

## Introduction

The past decades, extensive genomic analysis of tumor specimens using massive parallel sequencing by large sequencing consortia (e.g. https://www.icgc.org/icgc and http://cancergenome.nih.gov/) have revealed the major somatic drivers of human cancer, that have been reported in numerous studies. However, the small circular genome of the mitochondria has been largely ignored in such analyses. The human mitochondrial DNA (mtDNA) consists of ~16,569 base pairs encoding 37 genes: two rRNAs and twenty-two tRNAs functioning in the mitochondrial translation apparatus and thirteen proteins essential for oxidative phosphorylation. The total number of mtDNA molecules per cell varies between cell types from a few up to several thousand, and depends on both the number of mitochondria per cell as well as the number of mtDNA molecules per mitochondrion^1–3^. Similar to chromosomal DNA in the nucleus (nDNA), mtDNA may contain rare or polymorphic variants. Currently nearly 10,000 variable positions within mtDNA are reported in public databases^4^. When variation is acquired, genetically different mtDNA molecules can reside within a single cell, referred to as heteroplasmy (that is, >0% and <100% allele frequency per cell). Importantly, heteroplasmic patterns can differ within an individual across tissues^5–8^. Despite inherited and somatically acquired variants in mtDNA being associated with multiple human diseases^9^, the exact significance of somatic mtDNA variants in cancer remains controversial^10,11^.

Recently, taking advantage of publically available data from the large sequencing consortia, a handful of papers reported on the catalog of somatic mitochondrial variants in multiple tumor types^12–14^. However, a complicating issue in the genomic analysis of mtDNA is the presence of sequences of mitochondrial origin in the nDNA (termed nuclear insertions of mitochondrial origin, NUMTs). NUMTs have likely originated from joining mtDNA/RNA fragments to nDNA ends during double strand break repair^15,16^ and are found in nearly all eukaryotes that contain mtDNA. This process may occur at any moment during lifetime^17^ as well as during tumor evolution^18^. There are fixed NUMTs present in virtually every human genome–and thus reported in the human reference genome–inserted millions of years ago, but also more recent NUMT insertions have been described^19^. Unfortunately, due to their sequence similarity to mtDNA, NUMTs can interfere with accurate variant detection and thus investigation of mitochondrial heteroplasmy^16,19–23^. Estimations based on the human reference genome indicate that for each 175 base pairs mtDNA segment an average of 9.5 NUMT copies are present in the human nDNA^24^, but this number may likely be higher^19^. In addition, since the insertion of the mitochondrial genome is an ongoing process, this number is even larger in tumor cells since they also contain all somatic insertions events of NUMTs^18^. In addition, in tumor cells the processes shaping nDNA^25,26^ are substantially different from the one that shapes the mtDNA^13^, resulting in somatic variants in NUMTs and complicating accurate mtDNA heteroplasmy detection even further for tumor cells.

Consequently, the large variation in mtDNA between and within individuals as well as the presence of NUMTs demands a highly specific and sensitive detection of mtDNA variants, especially for low-frequent tumor-specific variants. In the study described here, we aimed to develop a sensitive procedure to detect low-frequent single-nucleotide mtDNA variants in frozen tumor tissue. Multiple efforts in developing methods for extraction of pure mtDNA exist^27–34^. These include methods using commercial kits or (laborious) ultracentrifugation to obtain pure mitochondria, and techniques to enrich for mtDNA by either the isolation technique or enzymatic degradation of nDNA. Unfortunately, the majority of previous studies focused on either cultured cells or cells from the blood and not on more physically and biochemically complex structures formed by tissue specimens. Thus, the application of these techniques to frozen tumor tissue specimens–an important source to assess tumor cell characteristics–has not been shown to date. Therefore, we compared four easily implementable procedures to extract mtDNA as pure as possible from frozen tumor tissue. Also, we evaluated three state-of-the-art techniques for the detection of low-frequent mtDNA-specific variants: Pacific Biosciences’ SMRT sequencing^35^, UltraSEEK chemistry^36^ and digital PCR.

## Results

### Procedure to obtain mtDNA-enriched DNA extracts from frozen tumor tissue

**Figure 1:**
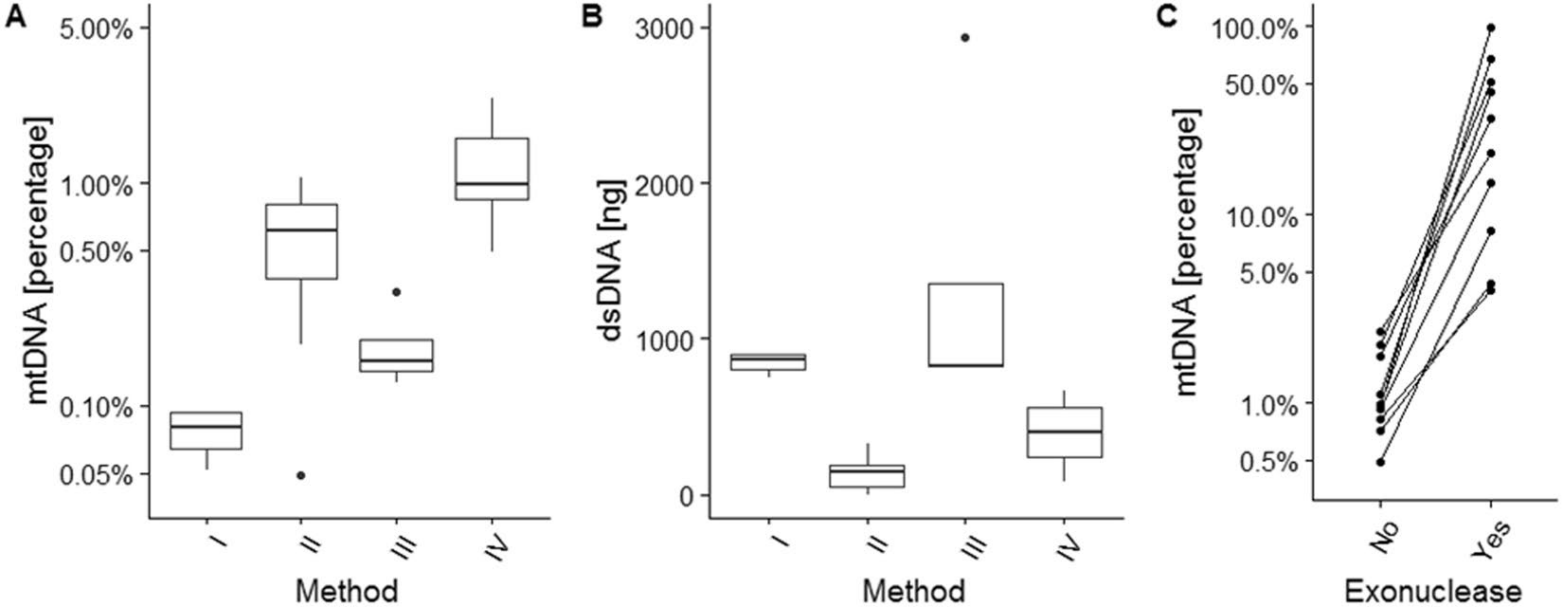
Comparison of methods for mtDNA extraction from fresh frozen tumor specimens. Cryosections originating from ten frozen tumor specimens (biological replicates) were subjected to different extraction procedures including (I) a total cellular DNA extraction method, (II) a method based on alkaline extraction, (III) a method extracting DNA from isolated mitochondria and (IV) a selective lysis method extracting DNA from cytosol fractions. For each method, the percentage of mtDNA (**A**) and total amount of dsDNA (**B**) was quantified. Also, DNA extracts from cytosol fractions originating from ten frozen tumor specimens were subjected to exonuclease-based enrichment and the percentage of mtDNA quantified, with for each specimen the mtDNA percentage before and after treatment connected by lines (**C**). Boxplots represent median, inter quartile range (IQR) and 1.5 × IQR.

To obtain mtDNA as pure as possible from frozen tumor tissue, our first focus was on the most optimal isolation procedure to extract mtDNA with minimal carry-over of nDNA. For this, we extracted DNA from fresh frozen primary tumor specimens using four easily implementable methods, and compared the yields via quantification of the percentage of mtDNA **(**Fig. 1A) and total amount of dsDNA **(**Fig. 1B). A silica-based total cellular DNA extraction method (I) used as reference for yield resulted in median 863 ng (interquartile range IQR 94 ng) dsDNA of which 0.1% (IQR 0.0%) mtDNA. A method (II) based on alkaline extraction–commonly used to extract plasmid DNA and thus designed to extract circular DNA^28,30,32,33^–yielded median 144 ng (IQR 140 ng) dsDNA with 0.5% (IQR 0.6%) mtDNA. Extracting DNA from isolated mitochondria (III)^34^ yielded median 825 ng (IQR 529 ng) dsDNA with 0.2% (IQR 0.1%) mtDNA. A selective lysis method (IV) that starts with the disruption of the plasma membrane to release the cellular components^29,37^ followed by sedimentation of cell nuclei, and DNA extracted from the remaining cytosol fraction yielded median 403 ng (IQR 321 ng) dsDNA with 1.0% (IQR 0.8%) mtDNA. Note that a similar trend was obtained by these methods using frozen cultured cells as input (Supplementary Figure 1A/B). From these results, it is evident that the best isolation procedure to extract mtDNA from frozen tumor tissue is method IV–DNA from cytosol fractions–with the highest mtDNA percentage and sufficient dsDNA yield. To increase the mtDNA fraction, we applied an enzymatic exonuclease reaction to degrade specifically linear nDNA. This greatly increased the percentage of mtDNA in DNA extracts from cytosol fractions, from median 1% (IQR 0.8%) to median 27% (IQR 40%) (Fig. 1C**)**. This result was also obtained when using DNA from frozen cultured cells as input material (Supplementary Figure 1C). Exonuclease treatment on total cellular DNA extracts increased the percentage of mtDNA as well, but not to the same extent as for DNA extracts from cytosol fractions, and total dsDNA yield was lower (Supplementary Figure 2). Concluding, the preferred procedure to obtain mtDNA as pure as possible from fresh frozen tumor tissue is to extract DNA from cytosol fractions followed by exonuclease treatment.

### Approach for sequencing of mtDNA

Next we explored sequencing methods for the detection of mtDNA variants. First, whole genome sequencing-by-synthesis (SBS) was applied to total cellular DNA extracts (method I) and DNA extracts from cytosol fractions (method IV), both without and with additional enrichment for mtDNA by exonuclease treatment. As expected, the cell line DNA extract from cytosol fraction treated with exonuclease yielded the highest percentage of aligned reads to mtDNA (86%), whereas the other methods yielded much lower percentages (<25%) (Supplementary Table 1**)**. The DNA extract from cytosol fraction treated with exonuclease derived from fresh frozen tumor tissue yielded a percentage of aligned reads to mtDNA in line with the PCR-based mtDNA percentage (respectively 12% and 10%). Thus, despite the relatively high fraction of 10% mtDNA, a major proportion of reads were derived from nuclear DNA. The observed spread in mtDNA percentage in exonuclease treated method IV extracts from frozen tumor tissue (Fig. 1C) will therefore lead to a variable proportion of mtDNA reads using whole genome SBS. To circumvent this variability, we decided to explore a targeted approach for sequencing mtDNA.

For this, nine primer sets covering the complete mtDNA were evaluated for their specificity to mtDNA, as *in silico* BLAST search showed that the primers did not match to known NUMT sequences in the reference genome. Specificity of the nine primer sets was confirmed by the absence of PCR products in two mtDNA-depleted cell lines (Supplementary Figure 3), allowing mtDNA-specific sequencing of the nine amplicons using single-molecule real-time (SMRT) sequencing. This method is able to generate long reads, covering each amplicon in a single read. To obtain an estimate of sequencing output and to evaluate variants detected by the whole genome SBS and targeted SMRT sequencing approaches, we compared for the two approaches the sequencing output of MDA-MB-231 DNA extracts from cytosol fraction treated with exonuclease. Whole genome SBS generated a total of 800,504 reads of 100 nucleotides (of which 87% duplicated reads) and after alignment resulted in an evenly distributed coverage of median 201x (IQR 2, range 13–404). The 2,727 reads of 1,738–2,836 base pairs by targeted SMRT sequencing displayed more variable coverage among the amplicons with median 282x (IQR 132, range 87–761) (Supplementary Figure 4). The more variable coverage in targeted SMRT sequencing was mainly due to regions where amplicons overlapped, causing an increase in coverage (Supplementary Figure 4). Both sequencing approaches detected all 29 positions with a documented alternative allele in MDA-MB-231 against rCRS at homoplasmic levels (>99% allele frequency). Also additional heteroplasmic variants were detected, with no major differences observed between the two sequencing approaches (Supplementary File). Given the lower output in read depth per number of generated reads by whole genome SBS sequencing–due to a loss of reads which map to the nuclear genome–and the risk of introducing NUMTs hampering downstream analysis, we continued sequencing experiments using the targeted SMRT sequencing approach.

### Sensitive detection of low-frequent mtDNA variants

To detect low-frequent single-nucleotide variants in mtDNA, we evaluated three approaches: SMRT sequencing, UltraSEEK chemistry and digital PCR. As a source of mtDNA we used breast cancer cell lines MDA-MB-231 and MCF-7. A total of respectively 29 and 13 variants alternative to rCRS have been documented in the mtDNA of MDA-MB-231 (also see above) and MCF-7, with a total of 28 positions containing a different allele between the two cell lines. To determine detection limits empirically, we prepared mixtures of the cell lines–considering MDA-MB-231 as the mutant variant–to generate samples with allele frequencies of 0%, 0.001%, 0.01%, 0.1%, 1% and 10% variant. The mixture samples were subjected to the three detection methods, and we evaluated their ability to detect the mutant variant. By SMRT sequencing, we obtained a median coverage of 4,060x per sample (IQR 4,842x, range 648–34,263x) (see Supplementary Table 2 for coverage per sample per amplicon). In the 0% variant allele sample (pure MCF-7), we confirmed all 13 positions with an alternative allele against rCRS^38^ at >95% allele frequency. At 5/28 positions known to be different between the two cell lines, heteroplasmic variants were observed in all mixture samples (Supplementary Table 3), prompting us to omit these positions in further analysis for limit of detection. Thus, we explored 23 positions by SMRT sequencing and confirmed all variant alleles, with a detection limit of 0.1% for 21 positions and a detection limit of 1% for 2 positions (Table 1 and Supplementary Figure 6A). The UltraSEEK method employs amplification of the region(s) of interest by PCR and subsequent detection of the variant(s)-of-interest via a single-base extension using chain terminators labeled with a moiety for solid phase capture, allowing enrichment of product, and identification of the product using matrix-assisted laser desorption/ionization time-of-flight mass spectrometry^36^. By UltraSEEK, we explored 7 positions and detected all variant alleles at those positions, with a detection limit of 0.1% for 5 positions and a detection limit of 1% for 2 positions (Table 1 and Supplementary Figure 6B). In digital PCR, a sample is partitioned into many individual parallel probe-based PCR reactions, each reaction contains either one target molecule or none, allowing a “yes” or “no” answer for the target molecule containing the mutant and wildtype allele in each reaction. By digital PCR 2 positions were evaluated for the variant allele, and one variant allele was detected ≥0.01% allele frequency and the other ≥0.1% allele frequency (Table 1 and Supplementary Figure 6C).

**Table 1:** Limit of detection for low-frequent mtDNA variants by SMRT sequencing, UltraSEEK and digital PCR.

Position	Mutant (MDA-MB-231)	Wildtype (MCF-7)	Limit of detection per method		
			SMRT	UltraSEEK	digital PCR
153	G	A	≥0.1%	na	na
195	C	T	≥0.1%	na	na
1719	A	G	≥0.1%	na	na
2706	G	A	≥0.1%	na	na
6221	C	T	≥1.0%	≥1.0%	na
6371	T	C	≥1.0%	≥0.1%*	na
6776	T	C	≥0.1%	na	na
7028	T	C	≥0.1%	na	na
8506	C	T	≥0.1%	≥1.0%	na
9966	G	A	≥0.1%	na	na
11719	A	G	≥0.1%	na	na
12084	T	C	≥0.1%	≥0.1%	na
12705	T	C	≥0.1%	na	na
13966	G	A	≥0.1%	≥0.1%	≥0.01%
14470	C	T	≥0.1%	≥0.1%	na
14766	T	C	≥0.1%	na	na
15310	C	T	≥0.1%	≥0.1%*	≥0.1%
15380	A	G	≥0.1%	na	na
16093	C	T	≥0.1%	na	na
16184	C	T	≥0.1%	na	na
16223	T	C	≥0.1%	na	na
16265	G	A	≥0.1%	na	na
16278	T	C	≥0.1%	na	na

Detection of the mutant variant allele (MDA-MB-231 genotype) in the lowest mutant fraction mixture indicated per position per method (empirical limit of detection). For the UltraSEEK and digital PCR method this was limited to respectively 7 and 2 positions due to requirement of generating dedicated PCR primers. na = not analyzed. *Detected in 1 out of 3 replicate samples.

### Detection of *de novo* mtDNA variants by SMRT sequencing

Since by SMRT sequencing the entire mtDNA was sequenced, we explored all alternative alleles that were called in the dataset of the six sample mixtures containing 0%, 0.001%, 0.01%, 0.1%, 1% and 10% mutant variant frequency. A total of 132 variants were called at 126 positions (some positions contained more than one alternative allele, Supplementary Table 3). Besides the documented homoplasmic variants for these two cell lines (35 variants, including the 28 differing alleles described above and 7 concordant alleles), 97 *de novo* variants were detected. Of those, 55 appeared as false positive calls in Integrative Genomics Viewer^39^ since they were associated with homopolymer regions or were in close proximity to homoplasmic alternative variants (Supplementary Figure 5). Of the remaining 42 *de novo* variants, the allele frequency ranged from 0.01% to 24.8% (Table 2). To evaluate if those *de novo* variants are true positive variants or potential false positives, we assessed their validation within the dataset: independent observations of a variant in multiple mixtures, or independent observations of a variant in overlapping regions of the sequenced amplicons. Of the 42 *de novo* variants, 20 were present in multiple mixtures, whereas 22 were present in one mixture only (Table 2). Also, 5 had been detected in the mutant-only sample (100% MDA-MB-231) that was sequenced at lower depth by both SMRT and SBS sequencing (see Supplementary File). Ten *de novo* variants were detected in overlapping regions of the sequenced amplicons, and thus represent two independent observations within one sample (Table 2). This resulted in 26 *de novo* variants that could be validated in our dataset, and thus true positive calls. A total of 16 *de novo* variants were detected in only a single amplicon in a single sample (Table 2), and can in theory be false positive calls (i.e. PCR errors or sequencing errors). These potential false positive variants had an allele frequency between 0.03% and 0.34%. Based on this, if validation of variants in either multiple samples or multiple amplicons is not possible, a conservative threshold on allele frequency for *de novo* variant detection of the SMRT sequencing approach would be ≥1.0% allele frequency.

**Table 2:** Allele frequency of the heteroplasmic *de novo* variants detected in six cell line mixtures by SMRT sequencing.

Position	Variant	Detected amplicon^a^	Phased genotype^b^	Cell line mixture (mutant fraction)					
				0%	0.001%	0.01%	0.1%	1%	10%
76	T	A, B	Wildtype	24.06*	24.75*	24.44*	24.75*	24.35*	21.01*
15806	A	A	Wildtype	7.09	7.29	7.07	7.12	7.58	6.22
1062	A	B	Wildtype	1.29	1.34	1.24	1.33	1.20	1.37
10085	T	F	Wildtype	0.60	0.86	0.87	0.84	0.74	0.70
7029	T	E	Wildtype	0.49	0.48	nc	0.37	nc	0.47
14644	T	I	Wildtype	0.30	0.38	0.24	0.23	0.41	0.36
14817	T	I	Wildtype	0.29	0.23	0.33	0.34	nc	nc
72	C	A, B	Wildtype	0.13*	0.12*	0.14*	0.14*	0.19*	0.08*
15897	A	A	Wildtype	0.12	0.09	0.16	0.10	nc	0.15
1398	C	B	Wildtype	0.08	nc	0.08	0.05	nc	nc
39	T	A, B	Wildtype	0.06*	0.06*	0.03*	0.10*	0.08*	0.04*
5031	A	D	Wildtype	0.14	nc	nc	nc	0.17	nc
14751	T	I	Wildtype	nc	0.15	nc	0.09	0.16	nc
15129	C	I, A	Wildtype	nc	0.05*	0.05*	nc	nc	nc
934	A	B	Wildtype	nc	0.05	0.05	nc	nc	nc
564	A	B	Wildtype	nc	0.05	nc	0.08	nc	nc
12124	T	G, H	Wildtype	nc	0.05*	nc	0.04*	0.07*	nc
103	A	A, B	Wildtype	nc	nc	nc	0.01*	nc	nc
13680	T	H, I	Wildtype	nc	nc	nc	0.03*	nc	nc
10607	T	F, G	Wildtype	nc	nc	nc	nc	0.06*	nc
16391	A	A	Wildtype	0.07^#^	nc	nc	nc	nc	nc
9808	T	F	Wildtype	nc	nc	0.08^#^	nc	nc	nc
11778	A	G	Wildtype	nc	nc	nc	0.06^#^	nc	nc
14607	A	I	Wildtype	nc	nc	nc	0.06^#^	nc	nc
228	A	B	Wildtype	nc	nc	nc	0.05^#^	nc	nc
9627	A	F	Wildtype	nc	nc	nc	0.04^#^	nc	nc
9804	A	F	Wildtype	nc	nc	nc	0.04^#^	nc	nc
15550	T	A	Wildtype	nc	nc	nc	0.03^#^	nc	nc
15604	T	A	Wildtype	nc	nc	nc	0.03^#^	nc	nc
16067	T	A	Wildtype	nc	nc	nc	0.03^#^	nc	nc
16169	T	A	Wildtype	nc	nc	nc	0.03^#^	nc	nc
664	A	B	Wildtype	nc	nc	nc	nc	0.09^#^	nc
12818	A	H	Mutant	nc	nc	nc	0.06^+^	0.91^+^	8.03^+^
16184	A	A	Mutant	nc	nc	nc	0.07^+^	0.41^+^	6.89^+^
763	A	B	Mutant	nc	nc	nc	0.06^+^	0.61^+^	6.16^+^
13623	T	H, I	Mutant	nc	nc	nc	nc	nc	0.31*^+^
10406	A	F, G	Mutant	nc	nc	nc	nc	nc	0.18*
6887	T	E	Mutant	nc	nc	nc	nc	nc	0.88^+^
3714	G	C	Mutant	nc	nc	nc	nc	nc	0.34^#^
16218	T	A	Mutant	nc	nc	nc	nc	nc	0.22^#^
3697	A	C	Mutant	nc	nc	nc	nc	nc	0.23^#^
1323	A	B	Mutant	nc	nc	nc	nc	nc	0.14^#^

^a^The amplicon (termed A to I) in which the variant was detected, which can be either one or two in the case of overlapping regions. ^b^The genotype of the variant as determined by allelic phasing (i.e. either MCF-7 considered wildtype or MDA-MB-231 considered mutant). nc = not called. *Variants that are detected in two overlapping amplicons and thus by two independent observations. ^+^Variants that were detected in a sample containing 100% mutant material by both SMRT and SBS sequencing at a lower depth (Supplementary File). ^#^Variants that can in theory be PCR errors because they were detected in only a single amplicon in a single sample.

### Allelic phasing of mtDNA variants detected by SMRT sequencing

**Figure 2:**
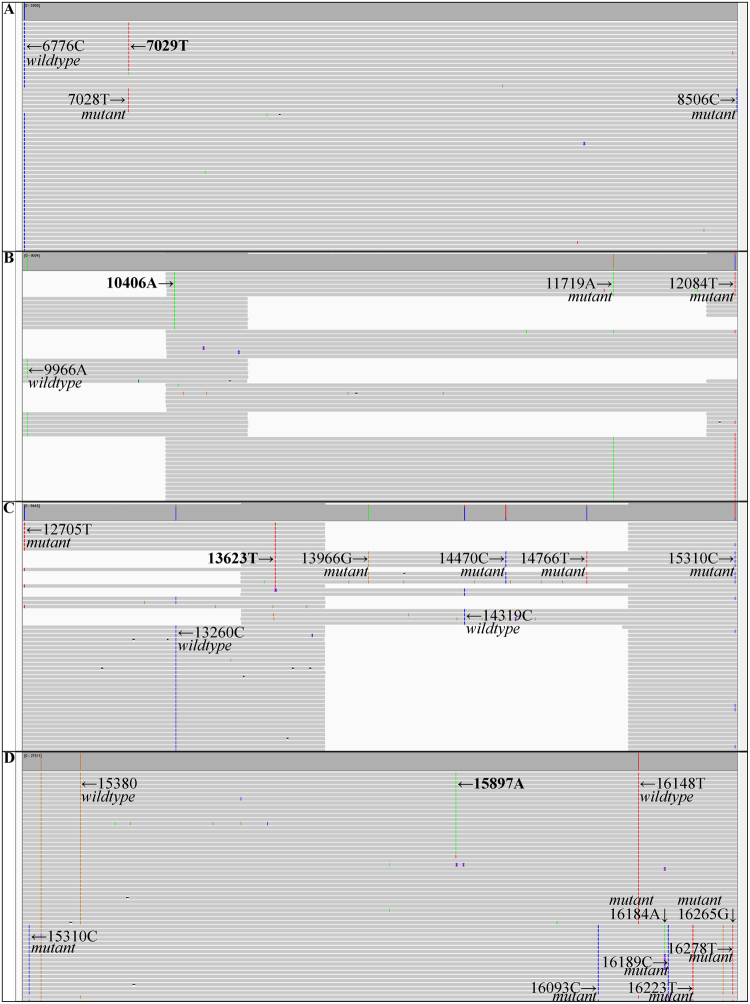
Phasing of *de novo* variants with variants known to belong to either the wildtype (MCF-7) or mutant (MDA-MB-231) genotype, exemplified by four Integrative Genomics Viewer (IGV) screenshots. (**A**) In the 0.1% mutant sample, position 7029 (T, red) phases together with reads containing the wildtype (MCF-7) variant at position 6776 (C, blue) but not the mutant (MDA-MB-231) variants at positions 7028 (T, red) and 8506 (C, blue). (**B**) In the 10% mutant sample, position 10406 (A, green) phases together with reads containing the mutant (MDA-MB-231) variants at position 11719 (A, green) and 12084 (T, red) but not the wildtype (MCF-7) variant at position 9966 (A, green). Note that position 10406 is covered by two amplicons, and thus detected by two independent observations. (**C**) In the 10% mutant sample, position 13623 (T, red) phases together with reads containing the mutant (MDA-MB-231) variants at position 12705 (T, red), 13966 (G, orange), 14470 (C, blue), 14766 (T, red) and 15310 (C, blue) but not the wildtype (MCF-7) variants at position 13260 (C, blue) and 14319 (C, blue). Note that position 13623 is covered by two amplicons, and thus detected by two independent observations. (**D**) In the 0.1% mutant sample, position 15897 (A, green) phases together with reads containing the wildtype (MCF-7) variants at position 15380 (G, orange) and 16148 (T, red) but not the mutant (MDA-MB-231) variants at position 15310 (C, blue), 16093 (C, blue), 16184 (A, green), 16189 (C, blue), 16223 (T, red), 16265 (G, orange) and 16278 (T, red). Horizontal is the DNA sequence, vertical the individual reads, and alignments sorted by base. Note that the position in IGV does not correspond to the rCRS position due to the use of an extended reference for alignment (see Materials and Methods). INDELs < 2 bases are hidden for clarity.

The long read length of SMRT sequencing enables to phase variants i.e. determine if they are present on the same read or on separate reads and thus if they originated from the same or another mtDNA molecule (Fig. 2). By this, we could evaluate if variants phased together with the known homoplasmic variants of the wildtype (MCF-7) or of the mutant (MDA-MB-231) genotype. Of the 42 *de novo* variants, a total of 32 variants phased together with the wildtype genotype and not with the mutant genotype (Table 2**)**. The variants with an allele frequency ≥0.5% in the wildtype-only mixture (0% mutant) were typically detected in all mixtures, whereas variants ≤0.5% allele frequency in the wildtype-only mixture were typically detected in the mixtures with only low mutant fractions (Table 2), hence the detection limit of the method. The remaining 10 *de novo* variants phased together with the mutant genotype and not with the wildtype genotype. Among those 10 variants that phased together with the mutant genotype, were the five that had also been detected in the mutant-only sample (100% MDA-MB-231) sequenced at lower depth by both SMRT and SBS sequencing (see Supplementary File). Also here, variants with a higher allele frequency in the mutant-only sample were typically detected in the mixtures with high mutant fractions (Table 2), hence the detection limit of the method. Thus, by SMRT sequencing we were able to evaluate the origin of the 42 *de novo* variants, phased to either the wildtype or mutant genotype (Table 2).

## Discussion

In this research, we aimed to develop a sensitive procedure to detect low-frequent single-nucleotide mtDNA variants from frozen tumor tissue. In assessing tumor cell characteristics, tissue specimens are an important source to detect tumor-specific variants. Especially when the focus is on low-frequent variants, frozen tissue is more suitable than formalin-fixed paraffin-embedded tissue since the latter is prone to deamination artefacts^40^. We started by establishing an extraction procedure to obtain mtDNA as pure as possible from frozen tumor tissue. The optimal method was DNA from cytosol fractions (method IV) treated with exonuclease, and resulted in a 270-fold mtDNA enrichment when compared to total cellular DNA extraction (27% versus 0.1% mtDNA yield, Fig. 1). The method based on alkaline extraction that is normally applied to extract plasmid DNA has also been described by others for preparation of mtDNA-enriched samples^28,30,32,33^. In line with the work by Quispe-Tintaya *et al*.^33^, we find for frozen cultured cells a good mtDNA enrichment compared to total cellular DNA extraction (158-fold, Supplementary Figure 1). However, application to frozen tumor tissue resulted in only a 5-fold mtDNA enrichment (Fig. 1) indicating that this method is less suited for frozen specimens. The method that extracts DNA from isolated mitochondria has also been described by others^34^, for which we find for frozen cultured cells similar mtDNA enrichment levels compared to total cellular DNA extraction (3-fold, Supplementary Figure 1). However, again for frozen tumor tissue we observe lower mtDNA enrichment (2-fold, Fig. 1). Note that, although the alkaline-based and mitochondria-based extraction methods were equivalent, different methods were applied to extract total cellular DNA in the above mentioned studies, and even among silica-based extraction methods mtDNA yield can be different^41,42^. Importantly, DNA from cytosol fractions either with or without exonuclease treatment compared to total cellular DNA extraction did also show better results for cultured cells (resp. 33-fold and 760-fold enrichment, Supplementary Figure 1). Thus, generally, extraction methods that significantly enrich for mtDNA from frozen cultured cells (and possibly also blood cells) do not guarantee a proper enrichment for mtDNA from frozen tissue.

A high fraction of mtDNA obtained within the DNA extract is vital to minimize the presence of NUMTs, which may lead to misinterpretation of mtDNA variants. Due to the variable number of mtDNA molecules per cell and the variable frequency of NUMTs, estimating the potential misinterpretation with NUMTs is difficult and unique for each position in each individual. Since the generation of NUMTs is an ongoing process^17–19^ estimating NUMT frequency is even more difficult for tumor cells since, they contain all private and all somatic NUMT events that have occurred during tumorigenesis and before that time. This is why we have chosen–and recommend–to analyze a mtDNA extract as pure as possible in SMRT sequencing. Exemplifying, in the case of 20x abundance of a NUMT (which is the case for numerous mtDNA regions^24^) in a cell type with 500 mtDNA molecules, it is possible to misinterpret the NUMT as a mtDNA variant with 8% heteroplasmy (2 × 20/500) in a total cellular DNA extract. Indeed, misinterpretation of non-identical mtDNA and NUMT positions is not a rare event and multiple examples have been highlighted in the literature^16,20–23^. Therefore, obtaining a high mtDNA fraction corresponds to obtaining a high number of mtDNA molecules as opposed to nDNA molecules, decreasing the variant allele frequency of the NUMTs, thus diminishing the likelihood for misinterpretation: a 270-fold increase in mtDNA for the example mentioned above would result in suppressing the NUMT variant to 0.03% heteroplasmy (2 × 20/270 × 500).

To detect low-frequent variants in mtDNA, we compared three state-of-the-art approaches. All three methods–SMRT sequencing, UltraSEEK, digital PCR–obtained 100% sensitivity at 1% variant allele frequency (Table 1). Specifically, SMRT shows a sensitivity of 100% at 1% allele frequency, 91% at 0.1% allele frequency and 0% at 0.01% allele frequency. SMRT sensitivity mainly depends on the read depth: positions 6221 and 6371 were sequenced less deep and had a detection limit of 1% (Supplementary Table 3). UltraSEEK shows a sensitivity of 100% at 1% allele frequency, 71% at 0.1% allele frequency and 0% at 0.01% allele frequency. Digital PCR shows a sensitivity of 100% at 0.1% allele frequency, of 50% at 0.01% allele frequency and 0% at 0.001% allele frequency. Notably, whereas UltraSEEK and digital PCR are limited to the positions chosen beforehand, the SMRT sequencing approach is able to evaluate the entire mtDNA. Since to date no mutational hotspot regions have been described for mtDNA in primary tumor specimens^12–14^, this is a valuable feature to study tumor-specific mtDNA variants. A limitation of all three methods is that they start with PCR amplification, and due to the large variation in mtDNA between and within individuals, primer binding sites can encounter variants that can bias PCR amplification. A whole genome sequencing method would enable a more unbiased approach, where a DNA sample is fragmented and subsequently sequenced independent of variants present in the sample. However–as shown by our results using whole-genome sequencing-by-synthesis (SBS)–this method requires deeper sequencing since a substantial part of the reads will be derived from nDNA. A bioinformatics approach would also be needed to filter reads originating from known NUMTs. In addition, the observed spread in mtDNA percentage in DNA extracts from frozen tumor tissue (Fig. 1) will lead to variability in the proportion of mtDNA reads between specimens when using a whole genome sequencing approach. This variability is likely due to biological variability in the number of mtDNA molecules within a cell or biochemical differences (e.g. fat or stromal content) between specimens, or due to technical variability in the multiplex qPCR assay. Samples with an extreme high mtDNA:nDNA ratio (and thus those greatly enriched for mtDNA) will have their mtDNA Ct value at the upper end whereas the nDNA Ct will be at the lower end, making the ratio estimation more variable because Ct estimations are less reliable. Also, the observed number of duplicated reads in SBS (87%) is within the expected range for single-end sequencing of the mitochondrial genome. Due to its small size, it contains only 16,569 starting positions for the 776,959 generated reads (Supplementary Table 1). When no variants or sequencing errors would be present within the reads, this would result in 97.9% of the reads appearing as duplicate reads. One could also use a targeted approach prior to SBS sequencing. Amplification of the complete mitochondrial genome in a single amplicon has been applied in SBS approaches, obtaining an error rate of 0.33% at a read depth of 20,000x^43^. Sequencing such an amplicon by SMRT is not feasible with the current chemistry, since it would require a read length >80,000 base pairs (5 passes of ~16,569 base pairs). Our targeted approach to amplify mtDNA by primer sets to generate amplicons between 1,700 base pairs and 3,000 base pairs does allow for high quality SMRT reads (≥5 passes to create a consensus sequence, minimizing sequencing errors) covering the complete amplicon, and simultaneously minimizes the risk of NUMT amplification (87% of known NUMTs are mtDNA fragments ≤1,500 base pairs^22^). In addition, the used primer sets did not generate an amplification product in mtDNA-depleted counterparts of two cell lines (Supplementary Figure 3) nor products by *in silico* BLAST, affirming that known NUMTs are unlikely to interfere. A drawback is that template amplification by PCR can introduce errors that may result in false positive calls. To decrease this, the PCR used a high fidelity polymerase (error rate of ~10^−7^) and the number of PCR cycles was limited (15 + 5 cycli). This would theoretically mean that 98.5–97.5% of the generated products per amplicon are entirely error-free, or that each product contains 0.02 random errors. By setting alternative allelic calls to at least 5 independent high-quality reads we intent to minimize calling PCR errors. An alternative would be to employ molecular barcodes prior to PCR amplification, which will allow tracing PCR duplicates and thus yield more confident calls of the original molecules. Note that five of the *de novo* variants detected by SMRT present in only a single sample appeared on two amplicons and are thus independent observations and unlikely to be PCR errors (Table 2). For the *de novo* variants that appear in only one sample on one amplicon (*n* = 16) we cannot rule out that they are not PCR errors, despite their phasing with a particular genotype (Table 2). All those were low-frequent variants (allele frequency between 0.03% and 0.34%). Thus, given the 100% sensitivity at 1% allele frequency, the SMRT approach is able to call variants reliable ≥1% allele frequency. To ascertain that variants below 1% allele frequency are true variants, validation is necessary by either independent re-sequencing (an additional sample, or in some cases in overlapping regions of amplicons within the same sample) or an orthogonal method. Both UltraSEEK and digital PCR prove suitable as orthogonal methods to confirm allelic calls, since they are both able to detect low-frequent variants. Analysis by UltraSEEK can be performed in multiplex (up to hundreds): the region(s) of interest are PCR amplified and subsequently the variant(s)-of-interest are detected via a single-base extension using chain terminators labeled with a moiety for solid phase capture, enrichment of product, and identification using matrix-assisted laser desorption/ionization time-of-flight mass spectrometry. However, both UltraSEEK and digital PCR are not suitable for *de novo* variant detection because they do need information on the variants-of-interest beforehand. Also, primer design has to be done for each variant separately, which can be limiting due to design constraints. The sensitivity of UltraSEEK mainly depends on the number of molecules analyzed, where 3 variant copies would suffice for detection (corresponding to at least 3,000 total copies for a 0.1% allele frequency). Analysis by digital PCR can be performed in multiplex (up to 4–8), with for each DNA molecule the region of interest is PCR-amplified and subsequently detected by specific probes on the variant-of-interest. Also in here, sensitivity mainly depends on the number of input molecules (minimal 2 variant copies of ≤20,000 total copies). The SMRT sequencing approach is as performant in terms of sensitivity (dependent on minimal 5 alternative reads) compared to these two methods, but is not limited to the necessity of knowing positions of variants-of-interest beforehand.

To conclude, our sensitive procedure to detect low-frequent single-nucleotide mtDNA variants from frozen tumor tissue is based on the extraction of DNA from cytosol fractions followed by exonuclease treatment to obtain high mtDNA yield, and subsequent SMRT sequencing for (*de novo*) detection and allelic phasing of variants. Orthogonal validation of variants can be done by either UltraSEEK (in the case of numerous variants) or digital PCR (in the case of a few variants). We conclude that the presented approach enables mtDNA-specific detection of *de novo* variants ≥1% allele frequency.

## Materials and Methods

### Specimens

Cell lines MDA-MB-231 and MCF-7 were cultured using RPMI (*Invitrogen*) supplemented with FBS (10%) (*Lonza*), 100 U/mL penicillin (*Invitrogen*), 100 µg/mL streptomycin (*Invitrogen*) and 0.05 mg/mL gentamycin (*Invitrogen*). A mtDNA-depleted MDA-MB-231 breast cancer cell line (MDA-MB-231-ρ0) was established by culturing MDA-MB-231 cells in the presence of 50 ng/µL ethidium bromide for 100 days in medium supplemented with uridine (0.05 mg/mL) (*Sigma-Aldrich*) and pyruvate (1 mM) (*Invitrogen)*. Frozen 143B and 143B-ρ0 osteosarcoma cell line pellets were kindly provided by dr. W.N.M. Dinjens (Department of Pathology, Erasmus MC). Fresh frozen primary breast tumor tissue specimens (resection material) were selected from the tumor biobank at the Erasmus MC (n = 10, stored in liquid nitrogen). The use of these patient materials was approved by the medical ethics committee of the Erasmus MC (MEC 02.953) and in accordance to the code of conduct of Federation of Medical Scientific Societies in the Netherlands. In the Netherlands, according to the Code of Conduct, informed consent is not required for retrospective analysis of bio-specimens retrieved during standard of care procedures.

### DNA extraction and mtDNA enrichment

Input for frozen tumor tissue was standardized at 20 cryosections of 30 µm thickness, which resulted in an average input of 19.2 mg (range of 5.9–33.4 mg) tumor tissue per extraction. Input for cultured cells was standardized at 1 million frozen cells per extraction. Total cellular DNA was extracted using the NucleoSpin Tissue kit (*Macherey-Nagel*) according to the supplier’s protocol (method I). Alkaline-based extraction was performed using the QIAprep Spin Miniprep kit (*Qiagen*), according to the supplier’s protocol (method II). Mitochondria were extracted using the Qproteome mitochondria isolation kit (*Qiagen*) according to the supplier’s protocol, and subsequently DNA was extracted using the NucleoSpin Tissue kit (above) (method III). To remove cell nuclei, samples were lysed using detergent that dissolves the cellular membrane (1 mL of 0.5x TBE containing 0.5% (v/v) Triton X-100^37^) for 10 minutes, followed by sedimentation of the nuclei at 1,020 × g for 10 minutes. From the remaining supernatant–the cytosol fraction–DNA was extracted using the QIAamp Circulating Nucleic Acid Kit (*Qiagen*) according to the suppliers’ protocol (method IV). In experiments to remove linear DNA, extracts (max. 100 ng DNA) were treated with 40 units of the ATP-dependent exonuclease PlasmidSafe (*Epicentre*) for 3 hours at 37 °C. Exonuclease was heat-inactivated (30 minutes 70 °C) and the circular DNA was purified using ethanol precipitation (70% ethanol).

### DNA quantification and mtDNA purity assessment

All DNA extracts were quantified using the Qubit dsDNA HS assay kit (*Life Technologies*) according to the suppliers’ protocol. Purity of mtDNA was assessed in duplicate runs of a multiplex qPCR assay targeting a nuclear and a mitochondrial encoded gene to calculate the ratio of mtDNA molecules opposed to nDNA molecules by the relative quantitation method (2^ΔCq^) as described before^44^. The percentage of mtDNA in the DNA extract was quantified (eq. 1) based on the ratio mtDNA:nDNA molecules and the sizes of the mitochondrial reference genome (16,569 base pairs, NC_012920) and complete reference genome (haploid 3,088,269,805 base pairs, GRCh38). If no amplification signal for the nuclear encoded gene was obtained, the ratio mtDNA:nDNA was set to 20,000,000 corresponding to a mtDNA percentage of 99%.

1 $$mtDNA\,percentage=\frac{{r}{a}{t}{i}{o}\ast {m}{i}{t}{o}{c}{h}{o}{n}{d}{r}{i}{a}{l}\,genome\,size}{({r}{a}{t}{i}{o}\ast {m}{i}{t}{o}{c}{h}{o}{n}{d}{r}{i}{a}{l}\,genome\,size)+nuclear\,genome\,size}\ast 100$$

### Whole genome sequencing-by-synthesis (SBS)

Input DNA was mechanically sheared using focused-ultrasonicator (*Covaris*) to yield fragments of ~300 base pairs in length, which required the following shearing-time for different DNA extracts: 90 seconds for total cellular DNA, 120 seconds for total cellular DNA treated with exonuclease, 90 seconds for cytosol fraction DNA, 50 seconds for cytosol fraction DNA treated with exonuclease. Sequence library was created using the Thruplex DNA-seq sample preparation kit (*Rubicon Genomics*), using 0.1–7.7 ng sheared input DNA. Sequencing was performed on an Illumina HiSeq2500 sequencer using HiSeq Rapid v2 chemistry and yielding 100 nucleotides single-end reads.

### UltraSEEK

UltraSEEK assays were designed using the AgenaCx online assay design software which automatically selects the PCR and extension primers (Supplementary Table 4), and adds to each reaction control assays for PCR and capturing. All oligonucleotides were obtained from Integrated DNA Technologies and control oligos from Agena Bioscience GmbH. Reactions were performed as described before^36^, using reagents obtained from Agena Bioscience. Briefly, PCR (45 cycles) was followed by shrimp alkaline phosphatase treatment and single base primer extension using biotinylated ddNTPs specific for the mutant alleles. After capture of the extended primers using streptavidin-coated magnetic beads, a cation-exchange resin was added for cleaning and 10-15 nl of the reaction was transferred to a SpectroCHIP® Array (a silicon chip with pre-spotted matrix crystals) using an RS1000 Nanodispenser (*Agena Bioscience*). Data were acquired via matrix-assisted laser desorption/ionization time-of-flight mass spectrometry using a MassARRAY Analyzer 4 (*Agena Bioscience*). After data processing, a spectrum was produced with relative intensity on the y-axis and mass/charge on the x-axis. Typer Analyzer software was used for data analysis and report generation.

### Digital PCR

Custom assays for two alternative variants were performed on the Quantstudio 3D digital PCR system (*Thermo Fisher*) according to the supplier’s protocol, with an adaption to the DNA input due to high mtDNA copy number. Reactions contained 20 pg of DNA in 1x dPCR mastermix v2, 0.9 µM of each primer (*Invitrogen*) and 0.2 µM of each probe (*Sigma*) (Supplementary Table 4). After initial denaturation for 10 minutes at 96 °C, the 40-cycle two-step PCR was performed at 30 seconds denaturation (98 °C) and 120 seconds annealing/extension (56 °C), and followed by a final 2 minute extension (56 °C). To calculate a variant frequency of the alternative variant, the threshold for signal dots was set to at least two dots.

### Single Molecule Real-Time (SMRT) sequencing

Amplicons covering the complete mtDNA^45,46^ (Supplementary Table 4) were generated in singleplex PCR reactions with initial denaturation for 3 minutes at 98 °C, 15 cycles of a three-step PCR with 10 seconds denaturation (98 °C), 30 seconds annealing (67 °C) and 90 seconds extension (72 °C), and final extension (72 °C) for 5 minutes. Each 50 µL reaction contained 2.5 ng of template DNA and 1 unit of Hot-Start Q5 High Fidelity DNA polymerase (*NEB*) in 1x Q5 reaction buffer, 200 µM dNTPs and 0.5 µM of each 5′-M13 tailed primer (*Invitrogen*) (Supplementary Table 4). Specificity of the generated products was confirmed using microchip electrophoresis (DNA-12000 reagent kit, *Shimadzu*). Amplicons were equimolar pooled per sample and purified using AMPure PB paramagnetic beads (*Pacific Biosciences*) with a 0.6 beads:sample ratio according to the SMRTbell Template Prep Kit protocol and eluted in 10 mM Tris-HCl pH 8.5. The 5′-M13 universal sequence tail of the primers allowed barcoding of each sample by performing 5 amplification cycles of the three-step PCR as described above but with an annealing temperature of 58 °C. Specificity of the generated products was confirmed using microchip electrophoresis (BioAnalyzer, DNA12000 or High Sensitivity DNA kit, *Agilent*). A final mix of barcoded fragments of all samples was obtained by equimolar pooling and subsequently purified using AMPure PB paramagnetic beads with a 0.6 beads:sample ratio. Concentration of the final mix was determined using the Qubit dsDNA HS assay kit, and SMRTbell library was generated according to the Amplicon Template Preparation and Sequencing guide (*Pacific Biosciences*). Sequencing was performed on Pacific Biosciences RSII with P6-C4 sequencing chemistry and 360 minutes movie-time or Sequel platforms with version 2 sequencing chemistry and 600 minutes movie-time. A total of twenty-two RSII and two Sequel SMRT cells were used to reach a read depth estimated at 3,000x per sample. In addition, two RSII SMRT cells were used to reach an estimated 5,000x for one sample (cell line mixture with 0.1% mutant allele frequency).

### Bioinformatics

Whole genome sequencing-by-synthesis (SBS) reads were trimmed and aligned using hisat2^47^ against the human reference genome GRCh38, after which the percentage of mtDNA was calculated (eq. 2). In addition, for evaluation of detected variants (Supplementary File), SBS reads were aligned against an extended version of rCRS (BWA-MEM version 0.7.15 default parameters^48^) and duplicate reads marked (Picard MarkDuplicates default parameters http://broadinstitute.github.io/picard/). We aligned the data against extended versions of rCRS (Supplementary Table 5) to compensate for mapping bias due to circularity of the mitochondrial genome.

2 $${percentage}\,{reads}\,{of}\,{mitochondrail}\,{origin}=\frac{{aligned}\,{reads}\,{on}\,{chrM}}{{aligned}\,{reads}\,{on}\,{GRCh38}}\ast 100$$

Single Molecule Real-Time (SMRT) sequencing RS bax.h5 files were converted to Sequel BAM files, of which circular consensus reads (CCS) were generated using the CCS2 algorithm for each sample-specific barcode^49^. Next, a minimum quality threshold of 99% and at least five passes of the SMRTbell were applied to select for highly accurate single-molecule reads. Selected CCS reads were trimmed (Cutadapt^50^ for primers-tails) and subsequently aligned against an extended rCRS (BWA- MEM version 0.7.15 parameters -k17 -W40 -r10 -A1 -B1 -O1 -E1 -L0^48^). We aligned the data against extended versions of rCRS (Supplementary Table 5) to compensate for mapping bias due to circularity of the mitochondrial genome.

For the comparison between SBS and SMRT sequencing methods (Supplementary File), pileup files were generated (Bioconductor Rsamtools 1.26.2 pileup function with pileupParam min_base_quality = 30, min_mapq = 0, min_nucleotide_depth = 0, min_minor_allele_depth = 0, distinguish_strands = TRUE, distinguish_nucleotides = TRUE, ignore_query_Ns = TRUE, include_deletions = FALSE, include_insertions = FALSE and in the case of SBS data flag isDuplicate = FALSE) and converted back to rCRS positions. For evaluation of detection limit and *de novo* variant detection for SMRT data, pileup files were generated as described above but with a more stringent threshold on the minimal number of alternative allele reads (min_nucleotide_depth = 5) to minimize detection of potential PCR errors (see Supplementary File). All detected variants were manually inspected in the Integrative Genomics Viewer (IGV, *Broad Institute*)^39^. Phasing of variants was done by manual inspection of every read containing the detected alternative variant and evaluating the other detected alternative variants present on that read.

MDA-MB-231 and MCF-7 mitochondrial sequences were obtained from the NCBI GenBank (resp. AB626609.1 and AB626610.1, deposited after resequencing by Imanishi *et al*.^38^) and blasted against rCRS to obtain the homoplasmic mtDNA positions alternative to the reference sequence for these two cell lines (NCBI’s nucleotide web blast, https://blast.ncbi.nlm.nih.gov).

### Data availability

Sequencing datasets can be accessed as BAM files (.bam) from the European Nucleotide Archive under accession number PRJEB23243.

## Electronic supplementary material

Supplementary Figures

Supplementary Tables

Supplementary Information File
