# Supplementary material for: Sensitive detection of mitochondrial DNA variants for analysis of mitochondrial DNA-enriched extracts from frozen tumor tissue
Source: Sci Rep. 2018 Feb 2;8:2261. doi: 10.1038/s41598-018-20623-7 (PMC5797170; doi:10.1038/s41598-018-20623-7)
Supplement: Supplementary file 1 — Supplementary Figures [file 41598_2018_20623_MOESM1_ESM.pdf]

TITLE

Sensitive detection of mitochondrial DNA variants for analysis of mitochondrial DNA-enriched extracts from frozen tumor tissue

AUTHORS AND AFFILIATIONS

M.J.A. Weerts[1*], E.C. Timmermans[2], R.H.A.M. Vossen[3], D. van Strijp[2], M.C.G.N. Van den Hout – van Vroonhoven[4], W.F.J. van IJcken[4], P.J. van der Zaag[2], S.Y. Anvar[3,5,6], S. Sleijfer[1], J.W.M. Martens[1]

1. Department of Medical Oncology and Cancer Genomics Netherlands, Erasmus MC Cancer Institute, Rotterdam, The Netherlands
2. Philips Research Laboratories, High Tech Campus 11, 5656 AE Eindhoven, The Netherlands
3. Leiden Genome Technology Center (LGTC), Department of Human Genetics, Leiden University Medical Center, Leiden, The Netherlands
4. Center for Biomics, Erasmus MC, Rotterdam, The Netherlands
5. Department of Human Genetics, Leiden University Medical Center, Leiden, The Netherlands
6. Department of Clinical Pharmacy and Toxicology, Leiden University Medical Center, Leiden, The Netherlands

SUPPLEMENTARY FIGURES 1 TO 6

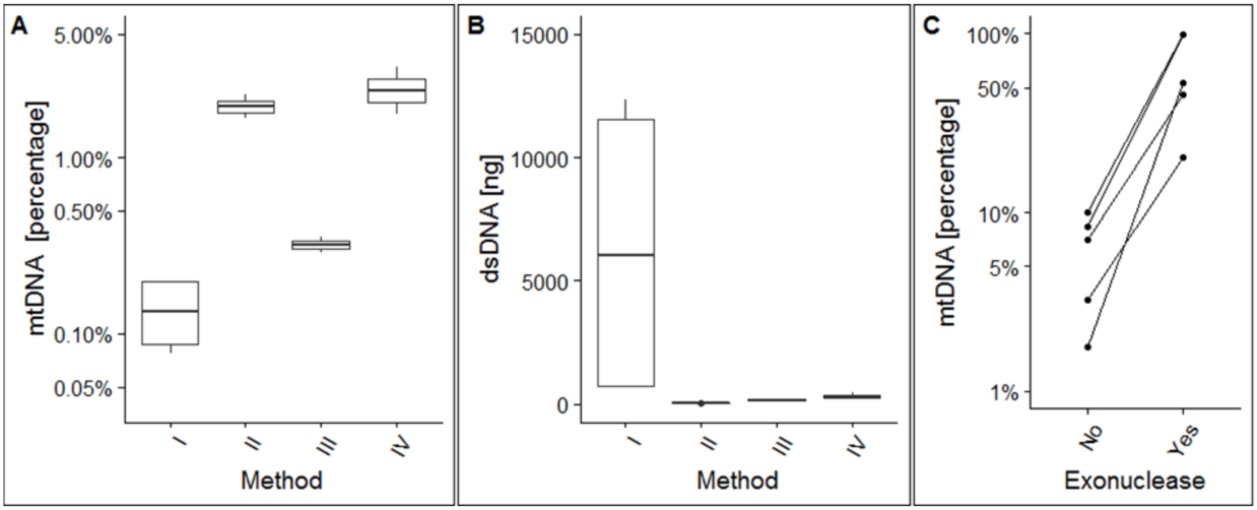

Supplementary Figure 1 Comparison of methods for mtDNA extraction from frozen MDA-MB-231 cells. Cells (one million per technical replicate) were subjected to different extraction procedures including (I) a total cellular DNA extraction method, (II) a method based on alkaline extraction, (III) a method extracting DNA from isolated mitochondria and (IV) a selective lysis method extracting DNA from cytosol fractions. For each method, the percentage of mtDNA (**A**) and total amount of dsDNA (**B**) was quantified. Also, DNA extracts from cytosol fractions were subjected to exonuclease-based enrichment and the percentage of mtDNA quantified, with for each specimen the mtDNA percentage before and after treatment connected by lines (B). Boxplots represent median, inter quartile range (IQR) and 1.5 × IQR.

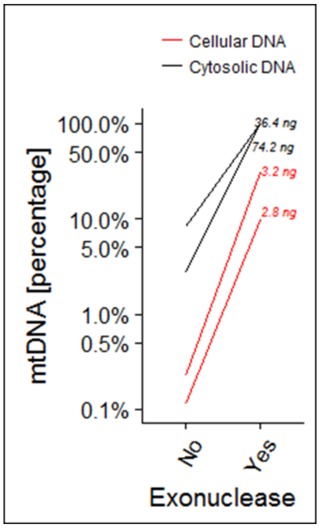

Supplementary Figure 2 Exonuclease-based enrichment for mtDNA in DNA extracts from frozen MDA-MB-231 cells. Total cellular DNA extracts (method I) (red) and cytosolic fraction DNA extracts (method IV) (black) were subjected to exonuclease-based enrichment (100 ng input per reaction) and yield was for the mtDNA percentage (vertical axis) and the total amount of dsDNA (text labels).

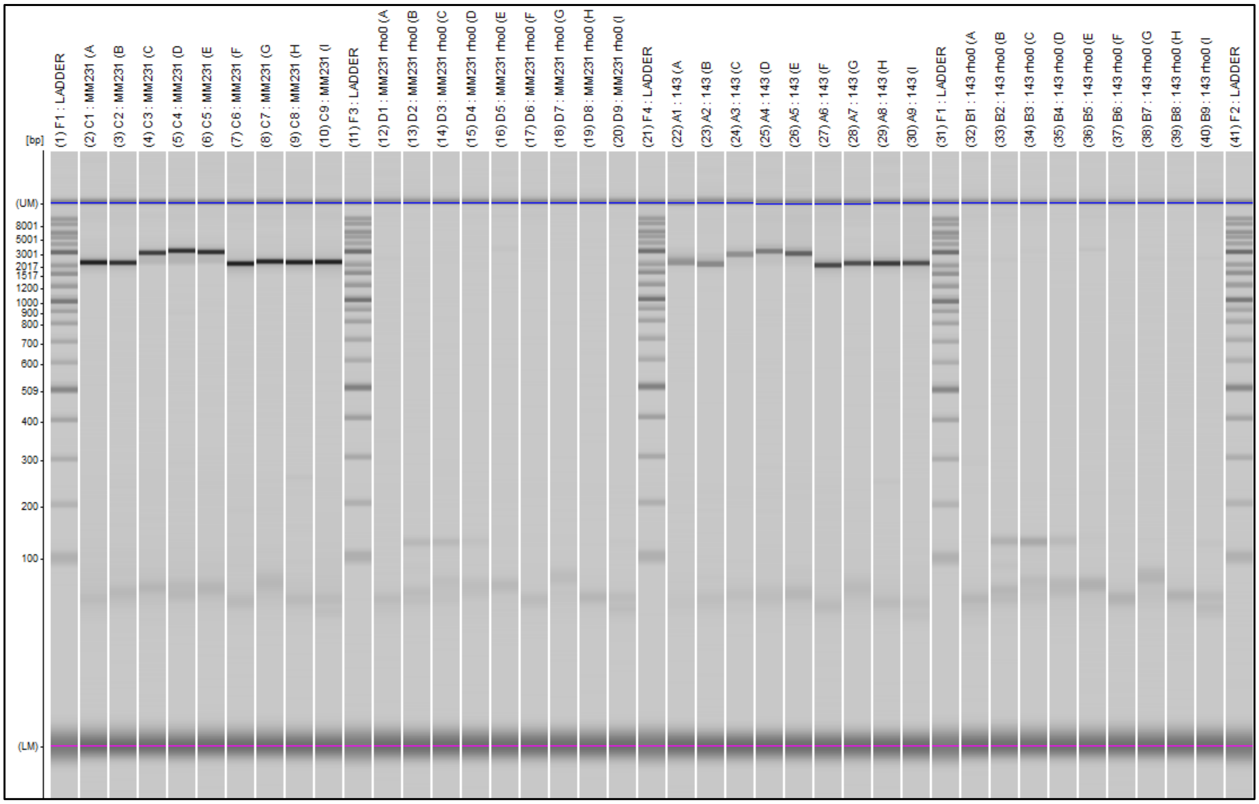

Supplementary Figure 3 Gel images (electropherogram analysis) after mitochondrial DNA amplification using the nine primer sets. Total cellular DNA extracts of cell lines MDA-MB-231 (mammary adenocarcinoma, human) and 143B (osteosarcoma, human) and their mtDNA depleted ρ0 counterparts were subject to amplification by each primer set (termed A to I) (horizontal columns). The ladders are reference for DNA fragment size, with base pairs (bp) indicated at the left border (vertical). UM upper marker, LM lower marker.

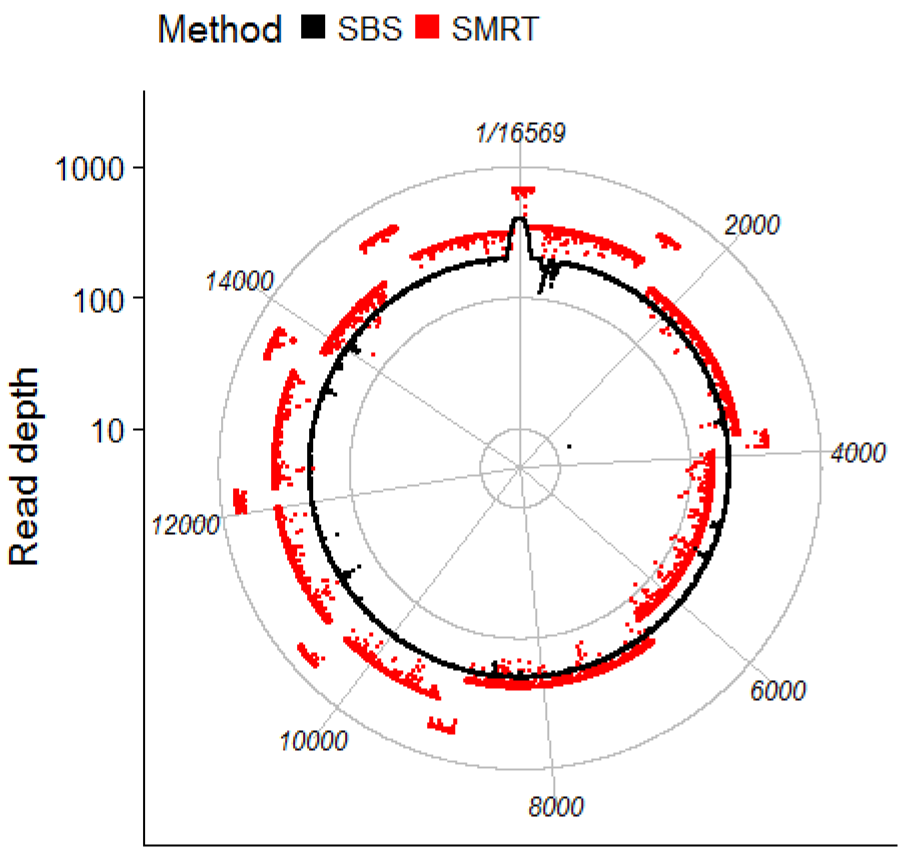

Supplementary Figure 4 Read depth of SBS and SMRT sequencing. Read depth (inner-to-outer) on the mitochondrial reference genome rCRS (circular) by SBS (black) and SMRT (red) sequencing of MDA-MB-231 cell line DNA extract from cytosolic fraction treated with exonuclease. The circle represents the mitochondrial genome and corresponding positions.

Note the increase in read depth by SBS at the mitochondrial D-loop region (between base pair positions 16450 to 150) due to the three-stranded DNA region [1], and the increase in read depth by SMRT sequencing at regions where two amplicons overlap.

1. Kasamatsu, H., D.L. Robberson, and J. Vinograd, *A novel closed-circular mitochondrial DNA with properties of a replicating intermediate.* Proc Natl Acad Sci U S A, 1971. **68**(9): p. 2252-7.

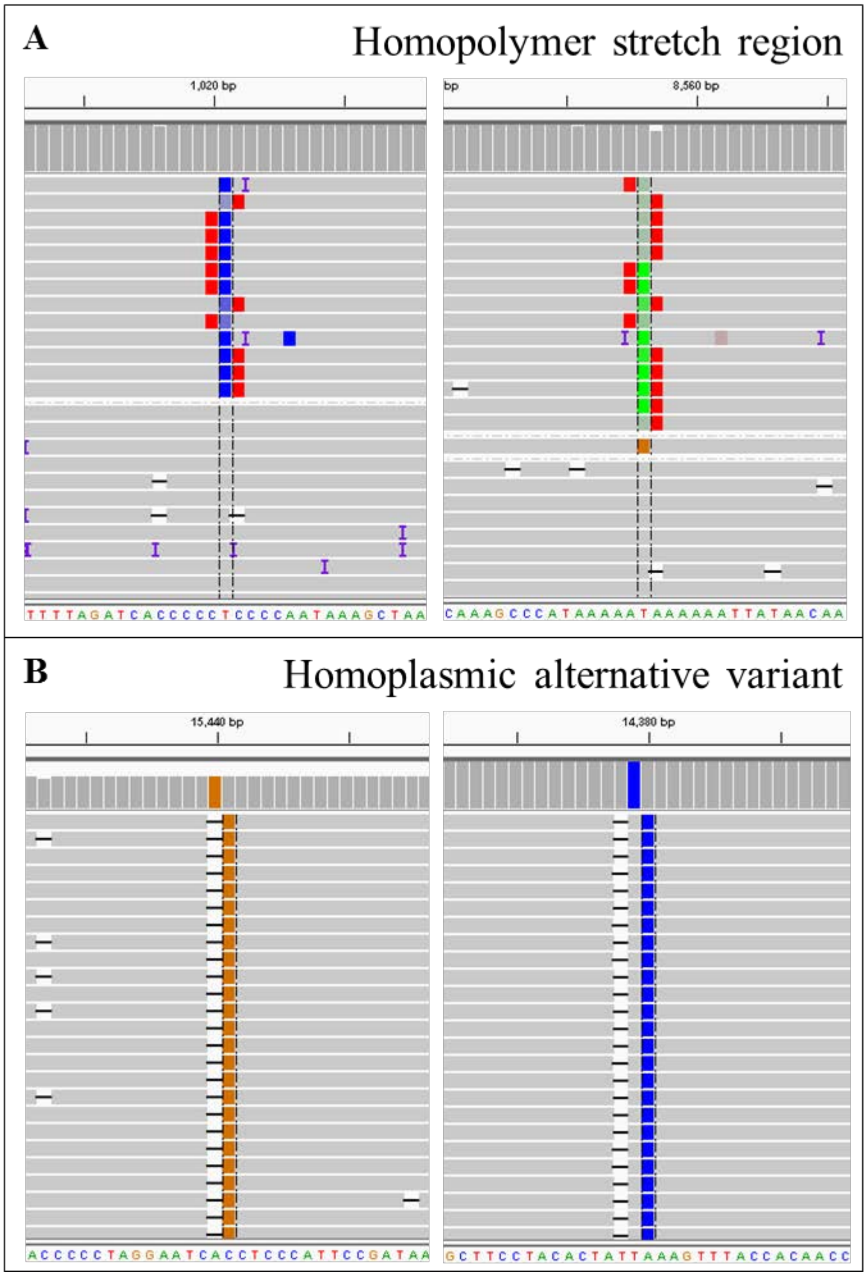

Supplementary Figure 5 Visualization (IGV) of examples of false positive calls observed in the SMRT sequencing datasets. The random but frequent insertions/deletions in SMRT data can difficult mapping. **A:** rCRS positions 960-962 (left) and 8496-8497 (right) due to a homopolymer stretch interrupted by a single variant (i.e. polyC-T-polyC / polyA-T-polyA). **B:** rCRS positions 15381 (left) and 14320 (right) due to an alternative polymorphism beside a homopolymer in the sample. Horizontal is the DNA sequence, vertical the individual reads, sorted by base. Note that the position in IGV does not correspond to the rCRS position due to the use of an extended reference for alignment (see Materials and Methods and Supplementary Table 2).

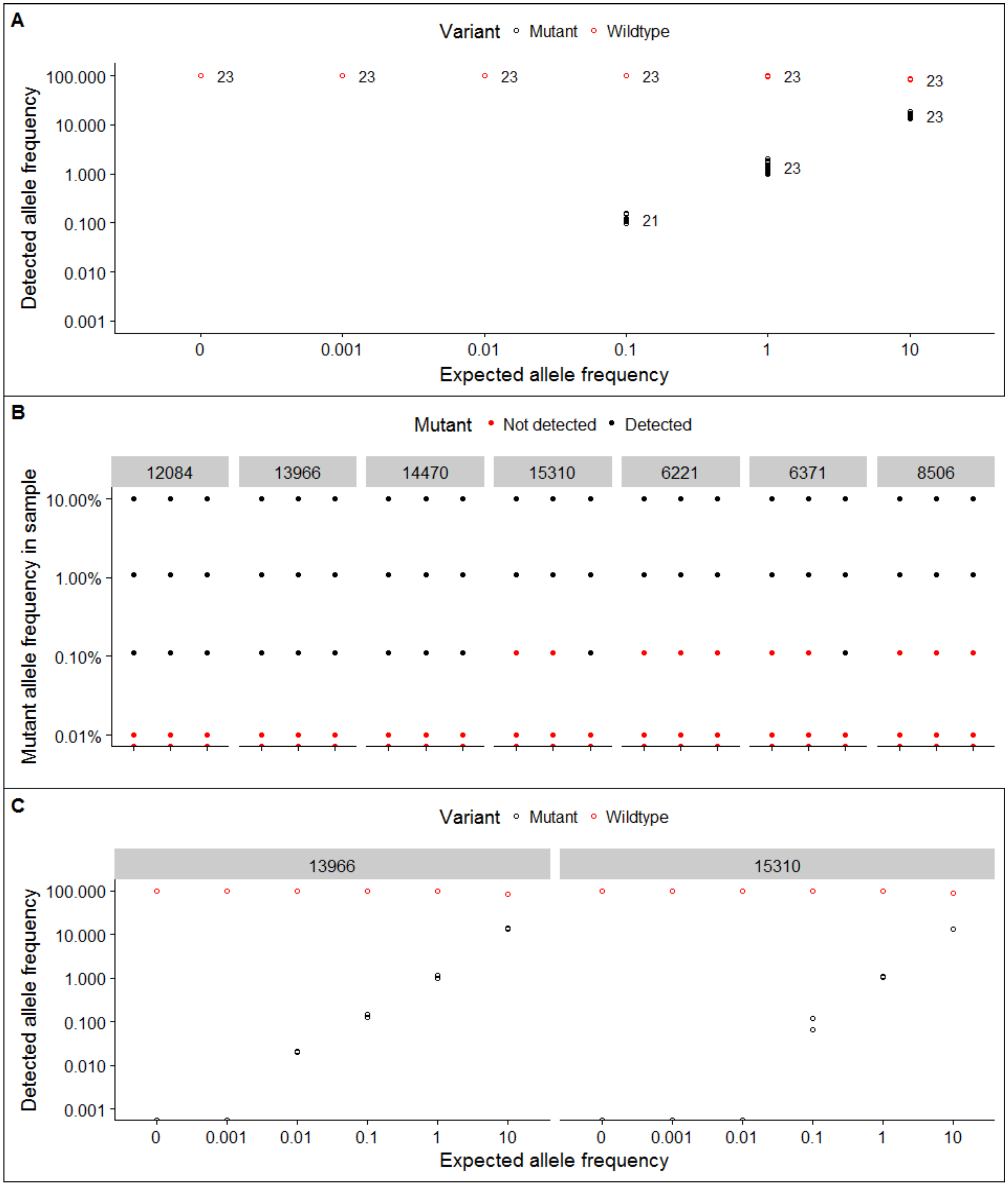

Supplementary Figure 6 Detection of mutant allele by SMRT sequencing, UltraSEEK and digital PCR in cell line mixtures containing 0, 0.001, 0.01, 0.1, 1 or 10% mutant allele frequency. A: SMRT sequencing. Indicated is the detected allele frequency (vertical) and the total number of positions (numbers) called as mutant (MDA-MB-231) (black) or wildtype (MCF-7) (red) allele per mixture (horizontal). **B**: UltraSEEK. Triplicate experiments, indicated is the total number of positive calls as mutant allele (MDA-MB-231) (black) or no call (red) for each mixture (vertical) at seven evaluated positions (horizontal panels). **C**: Digital PCR. Duplicated experiments, indicated is the detected allele frequency (vertical) called as mutant (MDA-MB-231) (black) or wildtype (MCF-7) (red) allele per mixture (horizontal).
