# Supplementary material for: Sensitive detection of mitochondrial DNA variants for analysis of mitochondrial DNA-enriched extracts from frozen tumor tissue
Source: Sci Rep. 2018 Feb 2;8:2261. doi: 10.1038/s41598-018-20623-7 (PMC5797170; doi:10.1038/s41598-018-20623-7)
Supplement: Supplementary file 3 — Supplementary Information File [file 41598_2018_20623_MOESM3_ESM.pdf]

TITLE

Sensitive detection of mitochondrial DNA variants for analysis of mitochondrial DNA-enriched extracts from frozen tumor tissue

AUTHORS AND AFFILIATIONS

M.J.A. Weerts[1*], E.C. Timmermans[2], R.H.A.M. Vossen[3], D. van Strijp[2], M.C.G.N. Van den Hout – van Vroonhoven[4], W.F.J. van IJcken[4], P.J. van der Zaag[2], S.Y. Anvar[3,5,6], S. Sleijfer[1], J.W.M. Martens[1]

1. Department of Medical Oncology and Cancer Genomics Netherlands, Erasmus MC Cancer Institute, Rotterdam, The Netherlands
2. Philips Research Laboratories, High Tech Campus 11, 5656 AE Eindhoven, The Netherlands
3. Leiden Genome Technology Center (LGTC), Department of Human Genetics, Leiden University Medical Center, Leiden, The Netherlands
4. Center for Biomics, Erasmus MC, Rotterdam, The Netherlands
5. Department of Human Genetics, Leiden University Medical Center, Leiden, The Netherlands
6. Department of Clinical Pharmacy and Toxicology, Leiden University Medical Center, Leiden, The Netherlands

SUPPLEMENTARY FILE

Comparison of variants detected by SBS and SMRT sequencing

Variants detected by SBS and SMRT sequencing of MDA-MB-231 DNA extracts from cytosol fraction treated with exonuclease were evaluated. After creation of pileup files – reporting all high-quality (99.9% accuracy) single-nucleotide calls at each position – all 29 positions with a documented alternative allele in MDA-MB-231 against rCRS [1] were detected by both approaches > 99% allele frequency. In addition, four high-frequent variants (≥ 5 % allele frequency) and eighteen low-frequent variants (< 5% allele frequency) were detected by both methods at comparable frequency, indicative that these are true heteroplasmic variants not documented previously (**Supplementary File Table 1**). Also, heteroplasmic positions were detected by either SMRT or SBS sequencing: 108 positions by SMRT and 1,030 positions by SBS. The majority of these variants were detected by low read depth of the alternative allele, indicative for false positives: by SMRT 105 variants with one alternative read (97.2% of all variants detected by SMRT), 2 variants with two alternative reads (1.9% of all variants detected by SMRT), and 1 variant with 21 alternative reads (0.9% of all variants detected by SMRT), whereas by SBS for 997 variants one alternative read was seen (96.8% of all variants detected by SBS), 29 variants with two alternative reads (2.8% of all variants detected by SBS), 1 variant with three alternative reads (0.1% of all variants detected by SBS), 1 variant with four alternative reads (0.1% of all variants detected by SBS), 1 variant with six alternative reads (0.1% of all variants detected by SBS), and 1 variant with 145 alternative reads (0.1% of all variants detected by SBS). The variants with one or two alternative reads (not shown) were not evaluated because the majority are likely false positive calls due to polymerase errors or sequencer errors [2]. The remaining heteroplasmic positions (>2 alternative reads) detected by either SBS or SMRT sequencing were evaluated and were likely false positives single-nucleotide calls as well: the variant detected by only SMRT at 21x alternative reads is adjacent to a SNP that likely complicates alignment (**Supplementary File Figure 1A**) and the variants detected by only SBS at 3x, 4x, 6x and 145x alternative read depth appear in either homopolymer stretches prone to PCR or sequencing errors, or were adjacent to position 3107 that likely complicates alignment (**Supplementary File Figure 1B/C/D**).

REFERENCES

1. Imanishi, H., K. Hattori, R. Wada, K. Ishikawa, S. Fukuda, K. Takenaga, K. Nakada, and J. Hayashi, *Mitochondrial DNA mutations regulate metastasis of human breast cancer cells.* PLoS One, 2011. **6**(8): p. e23401.
2. Goodwin, S., J.D. McPherson, and W.R. McCombie, *Coming of age: ten years of next-generation sequencing technologies.* Nat Rev Genet, 2016. **17**(6): p. 333-51.

Supplementary File Table 1 Positions of alternative variants detected in both the SBS and SMRT sequencing data of MDA-MB231 DNA extracts (cytosol fractions treated with exonuclease).

Position on mtDNA	Nucleotide		Documented in AB626609	Whole genome SBS data			Targeted SMRT data		
	Reference	Alternative		Total depth	Alternative depth	Alternative allele frequency	Total depth	Alternative depth	Alternative allele frequency
73	A	G	Yes	355	355	100.0	663	663	100.0
153	A	G	Yes	199	199	100.0	349	348	99.7
195	T	C	Yes	200	200	100.0	348	348	100.0
251	G	A	No	199	1	0.5	352	3	0.9
263	A	G	Yes	199	198	99.5	353	353	100.0
302	A	C	No	176	3	1.7	321	1	0.3
709	G	A	Yes	201	201	100.0	353	353	100.0
750	A	G	Yes	202	202	100.0	325	325	100.0
763	C	A	No	202	85	42.1	327	120	36.7
1438	A	G	Yes	198	197	99.5	602	602	100.0
1719	G	A	Yes	201	201	100.0	245	245	100.0
2706	A	G	Yes	200	199	99.5	248	248	100.0
2772	C	T	No	201	2	1.0	234	5	2.1
2900	C	T	No	202	1	0.5	242	1	0.4
2977	G	A	No	202	2	1.0	231	1	0.4
3107	N	C	No	199	195	98.0	250	250	100.0
3317	C	A	No	201	1	0.5	245	1	0.4
3352	G	A	No	202	2	1.0	249	1	0.4
3665	G	A	No	201	1	0.5	250	1	0.4
3903	C	T	No	199	1	0.5	394	1	0.3
4769	A	G	Yes	202	201	99.5	148	148	100.0
5985	G	A	No	202	2	1.0	148	2	1.4
6132	G	A	No	201	1	0.5	148	1	0.7
6221	T	C	Yes	200	200	100.0	125	125	100.0
6290	C	T	No	201	11	5.5	143	14	9.8
6371	C	T	Yes	198	198	100.0	148	148	100.0
6887	C	T	No	200	4	2.0	242	10	4.1
7028	C	T	Yes	199	199	100.0	240	240	100.0
8506	T	C	Yes	201	201	100.0	241	241	100.0
8860	A	G	Yes	201	201	100.0	240	240	100.0
10009	G	A	No	201	2	1.0	370	1	0.3
10969	C	T	No	193	1	0.5	370	7	1.9
11719	G	A	Yes	202	202	100.0	377	377	100.0
11771	C	T	No	201	3	1.5	378	1	0.3
12084	C	T	Yes	202	202	100.0	722	721	99.9
12705	C	T	Yes	199	199	100.0	369	369	100.0
12818	G	A	No	201	88	43.8	374	167	44.7
13219	C	A	No	202	1	0.5	375	1	0.3
13623	C	T	No	201	3	1.5	654	5	0.8
13966	A	G	Yes	201	201	100.0	274	273	99.6
14470	T	C	Yes	202	202	100.0	245	244	99.6
14766	C	T	Yes	201	201	100.0	274	273	99.6
15310	T	C	Yes	202	202	100.0	595	594	99.8
15326	A	G	Yes	200	200	100.0	321	321	100.0
16093	T	C	Yes	197	197	100.0	315	315	100.0
16184	C	A	No	198	144	72.7	235	159	67.7
16189	T	C	Yes	184	183	99.5	268	268	100.0
16223	C	T	Yes	199	199	100.0	316	316	100.0
16265	A	G	Yes	202	202	100.0	312	312	100.0
16278	C	T	Yes	198	198	100.0	317	317	100.0
16519	T	C	Yes	391	391	100.0	662	662	100.0

Note that variants detected by either SBS or SMRT sequencing are not shown.

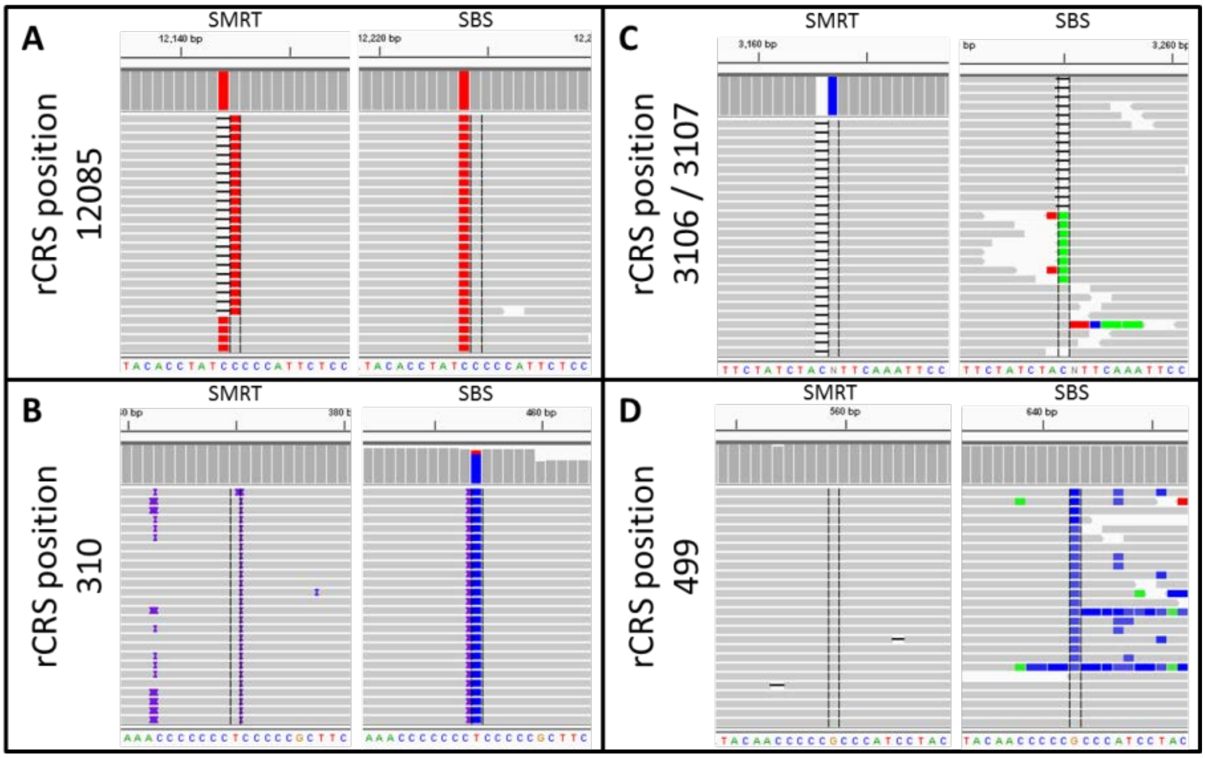

Supplementary File Figure 1 Alternative variants detected in either SBS or SMRT sequencing data with > 2 alternative reads.

Visualization (IGV) of the alleles at reference sequence (rCRS) position 12,085 (**A**), 310 (**B**), 3106 and 3107 (**C**), and 499 (**D**) with > 2 alternative reads detected by either only SMRT (left in each panel) or SBS (right in each panel) sequencing. Horizontal is the DNA sequence, vertical the individual reads, and alignments sorted by base (T in red, C in blue, A in green, G in orange). Note that the position in IGV does not correspond to the rCRS position due to the use of an extended reference for alignment (see Materials and Methods and Supplementary Table 2).
